# RNA-Seq de Novo Assembly and Differential Transcriptome Analysis of Chaga (*Inonotus obliquus*) Cultured with Different Betulin Sources and the Regulation of Genes Involved in Terpenoid Biosynthesis

**DOI:** 10.3390/ijms20184334

**Published:** 2019-09-04

**Authors:** Narimene Fradj, Karen Cristine Gonçalves dos Santos, Nicolas de Montigny, Fatima Awwad, Yacine Boumghar, Hugo Germain, Isabel Desgagné-Penix

**Affiliations:** 1 Department of Chemistry, Biochemistry and Physics, Université du Québec à Trois-Rivières, 3351, boul. des Forges, C.P. 500, Trois-Rivières, Québec, QC G9A 5H7, Canada; 2 Centre d’étude des Procédés Chimiques du Québec, 6220 rue Sherbrooke Est, Montréal, Québec, QC H1N 1C1, Canada; 3 Groupe de Recherche en Biologie Végétale, Université du Québec à Trois-Rivières, 3351, boul. des Forges, C.P. 500, Trois-Rivières, Québec, QC G9A 5H7, Canada

**Keywords:** chaga, de novo transcriptome, *Inonotus obliquus*, biosynthesis, terpenoid, betulinic acid, specialized metabolism, RNA-Seq

## Abstract

Chaga (*Inonotus obliquus*) is a medicinal fungus used in traditional medicine of Native American and North Eurasian cultures. Several studies have demonstrated the medicinal properties of chaga’s bioactive molecules. For example, several terpenoids (e.g., betulin, betulinic acid and inotodiol) isolated from *I. obliquus* cells have proven effectiveness in treating different types of tumor cells. However, the molecular mechanisms and regulation underlying the biosynthesis of chaga terpenoids remain unknown. In this study, we report on the optimization of growing conditions for cultured *I. obliquus* in presence of different betulin sources (e.g., betulin or white birch bark). It was found that better results were obtained for a liquid culture pH 6.2 at 28 °C. In addition, a de novo assembly and characterization of *I. obliquus* transcriptome in these growth conditions using Illumina technology was performed. A total of 219,288,500 clean reads were generated, allowing for the identification of 20,072 transcripts of *I. obliquus* including transcripts involved in terpenoid biosynthesis. The differential expression of these genes was confirmed by quantitative-PCR. This study provides new insights on the molecular mechanisms and regulation of *I. obliquus* terpenoid production. It also contributes useful molecular resources for gene prediction or the development of biotechnologies for the alternative production of terpenoids.

## 1. Introduction

*Inonotus obliquus*, a member of the Hymenochaetaceae family of Basidiomycetes, is a medicinal fungus used in the traditional medicine of Native American and North Eurasian cultures. *I. obliquus*, commonly known as chaga or black diamond, is used by some Canadian First Nations to treat infections and tumors. Chaga is a black parasitic fungus that grows on the living trunks of the northern latitudes of America, Europe and Asia, and it is the primary pathogen of birch and other Betulaceae trees [1]. In nature, instead of a fruiting body, it usually forms an irregular shape of a sclerotial conk with the appearance of burnt charcoal, and it has been used for centuries as a folk medicine to treat and prevent multiple diseases. Over the last decade, studies have revealed that the extract of *I. obliquus* contains biologically active molecules derived from the specialized metabolism, supporting the effectiveness of chaga in traditional medicine. These specialized metabolites (e.g., polysaccharides, polyphenols and terpenoids) are responsible for *I. obliquus*’s medicinal effects including those of the antioxidant [1,2,3,4,5,6,7,8,9,10,11], antibacterial [1,7,12,13], anti-diabetic [14,15,16,17,18,19], and anticancer [16,19,20,21,22,23,24,25,26,27,28,29,30,31,32,33,34,35,36,37] types.

Terpenoids consist of a large and diverse class of chemicals among the multitude of metabolites produced by fungi. Fungi employ terpenoids for an array of primary functions (growth and development) and for specialized activities, chemical interactions, and protection against abiotic and biotic factors. *I. obliquus* produces a diverse range of bioactive terpenoids exhibiting compelling therapeutic activity (e.g., anti-tumor, anti-inflammatory). Among the bioactive terpenoids found in chaga, sesquiterpenoids (e.g., bergamotene, selinene, and santalene) and triterpenoids (e.g., betulin, betulinic acid, lanosterol, inotodiol, and trametenolic acid) have been identified [1,35,38,39,40,41,42,43].

In addition, *I. obliquus* is able to catabolize metabolites from its birch host to grow and develop. White birch bark contains large amount of triterpenoids including betulin and betulinic acid, which represent up to 75% and 2% of the extractives, respectively [44,45]. It is not clear if betulin and betulinic acid detected in *I. obliquus* sclerotia result only from fungal de novo synthesis, from the transformation of birch terpenoid metabolites, or from both. It has been shown that *I. obliquus* cells are able to produce these triterpenoids in a submerged culture, suggesting the presence of genes encoding biosynthetic enzymes involved in their de novo production [46]. However, the identification and regulation of these genes remain unknown.

In fungi, terpenoid biosynthesis occurs via the mevalonate (MVA) metabolic pathway which can be divided into three parts (Appendix A—Figure A1). The first part (precursor pathway) involves a series of enzymatic reactions converting acetyl-CoA to isopentenyl diphosphate (IPP) and dimethylallyl diphosphate (DMAPP), which are the precursors of all terpenoids. For the second part (the sesquiterpenoid pathway), the cytosolic farnesyl diphosphate synthase (FPS) condenses two molecules of IPP with one molecule DMAPP head-to-tail sequentially to produce farnesyl diphosphate (FPP). FFP serves as a precursor for sesquiterpenoids, which are synthesized by terpene synthases and can be processed by other assorted enzymes. Lastly, the third part of the pathway involves reactions that catalyze the formation of three groups of triterpenoids: The amyrin-type, the lanosterol-type and the lupeol-type.

In recent years, high throughput sequencing platforms such as the Illumina RNA-Seq have been developed to study the transcriptome of model and non-model species, including plants, animals and microorganisms. This advanced sequencing technology has proven to be a cost-effective, rapid and powerful tool for investigating new functional genes and for detecting differentially expressed genes in diverse species. Currently, there is no genome information available for the Inonotus species; hence, the molecular basis of terpenoid production has not yet been elucidated. The aim of this study was to use de novo transcriptomic to generate a comprehensive profile of the genes related to terpenoid production in an *I. obliquus* cell culture supplemented or not with terpenoid substrates. Moreover, a better understanding of the metabolic pathway for the synthesis of *I. obliquus* pharmaceutically-relevant metabolites such as betulinic acid, betulin and inotodiol will enable tools to increase the production of these valued metabolites. Chaga’s host, i.e., white birch (*Betula papyrifera*), also produces betulin in high concentrations [47,48]. It has been reported that the use of microorganisms isolated from the same sources as the host substrate would increase the probability of obtaining derivatives of this substrate using the bioconversion approach [49]. Related to that, the co-culture of chaga with white birch bark residues could increase the conversion of betulin trapped in birch bark into betulinic acid to valorize forestry residues on one side, and, on the other side, it could elucidate the relationships between chaga and white birch bark.

In this study, the optimal growth conditions of *I. obliquus* cultured with or without betulin or white birch bark were investigated, and RNA was isolated. A cDNA library was then generated, and a whole transcriptomic analysis was performed using the Illumina HiSeq 4000. Whole-transcriptome was assembled de novo and analyzed, and differential expression studies confirmed by qRT-PCR unveiled the genes responsible for the terpenoid pathways in chaga.

## 2. Results and Discussion

### 2.1. Optimal Growth Conditions

**Figure 1:**
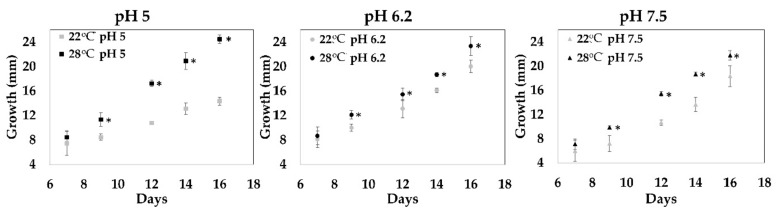
Effect of pH and temperature on *Inonotus obliquus* growth. *I. obliquus* was grown on a yeast malt agar medium (YMA) in Petri dishes for 16 days at 22 or 28 °C. YMA pH was adjusted with addition of an HCl or NaOH solution to obtain final pHs of 5, 6.2 and 7.5. Diameter (in mm) corresponding to fungal growth was measured every two days starting at day seven. Results represent the average (± error bars) growth of three biological repetitions per condition. Statistical significance is annotated with an asterix (*), according to ANOVA test results with *p* ˂ 0.05.

The sclerotia of *I. obliquus* grow very slowly in nature, and artificial culture is difficult. To produce large amounts of biomass, *I. obliquus* was cultured using different growth conditions. Mycelial growth tests were carried out on a culture medium to determine optimal conditions. The growth of the *I. obliquus* mycelium was conducted under nine conditions (three different pH (5, 6.2 and 7.5) and three different temperatures (22, 28, and 37 °C) over 16 days in a solid medium. Figure 1 shows the results for two temperatures because at 37 °C, no growth of *I. obliquus* mycelium was observed (data not shown). At 28 °C, *I. obliquus* growth was significantly higher for all tested pH; however, pHs 5 and 6.2 showed the best results (Figure 1). The effect of temperature on chaga’s growth was only observed at pH 5. For example, on day 16 at pH 5 in the solid culture, fungal growth at 28 °C was twice the growth observed at 22 °C (Figure 1; from 12 mm at 22 °C to more than 24 mm at 28 °C). However, the effect of pHs 5 or 6.2 on fungal growth at 28 °C was not significantly different (Figure 1; circa 24 mm for both pH).

**Figure 2:**
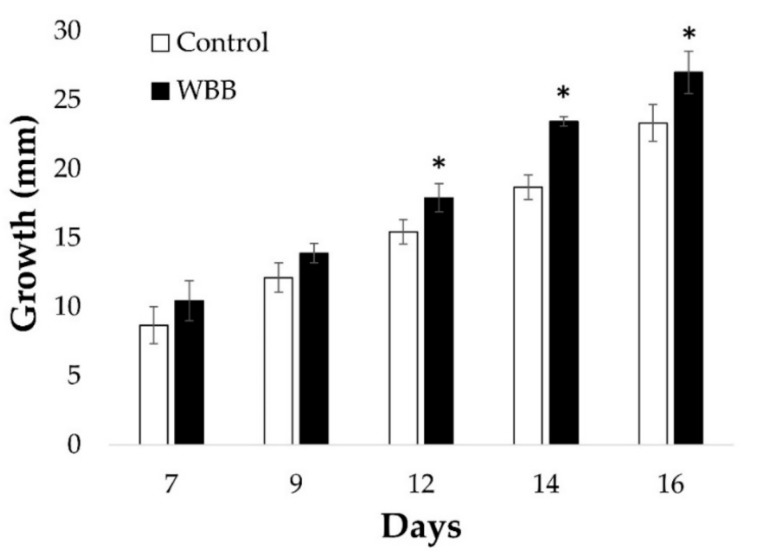
*Inonotus obliquus* cell culture growth in presence of white birch bark. *I. obliquus* was grown on a solid medium pH 6.2 at 28 °C for 16 days on agar plates containing a yeast malt agar (Control) medium (white bars) or YMA supplemented with white birch bark (WBB) fragments (black bars). The measurements of the diameter (mm) of growth were taken every two days starting at day seven. Results show the average (± error bars) growth of three biological repetitions per condition. Statistical significance is annotated with an asterix (*) according to ANOVA test results with *p* ˂ 0.05.

In the wild, the growth and production of terpenoids by *I. obliquus* is likely influenced by substrates provided by its host. Thus, an investigation to determine the influence of white birch bark (WBB) on growth of *I. obliquus* under optimal pH and temperature conditions was carried out. For this purpose, *I. obliquus* cells were grown in presence or absence of WBB residues. Results showed that *I. obliquus* growth was significantly higher in presence of WBB (Figure 2). Specifically, after 16 days of co-culture, the *I. obliquus* control mycelia reached 23 mm diameter, whereas those that were WBB-treated amounted to 27 mm (Figure 2). This indicates that *I. obliquus* growth was stimulated by the presence of bark residues from its host.

**Figure 3:**
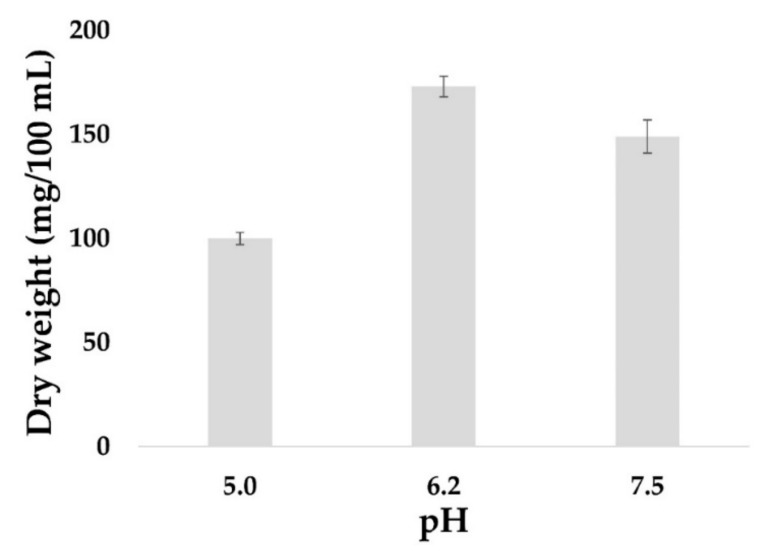
Effect of pH on *Inonotus obliquus* biomass. *I. obliquus* was grown in a liquid medium of a yeast malt broth medium (YMB) at 28 °C for eight days. The pH of the medium was adjusted by adding HCl or NaOH to obtain three final pH values of 5, 6.2 and 7.5. At day eight, *I. obliquus* cells were collected, filtered and dried at 70 °C overnight. Results represent the average (± error bars) dry weight (mg) of three biological repetitions per condition.

The yield of fungal biomass is often higher in a liquid culture compared to a solid culture. Furthermore, a liquid culture is easier to manipulate in order to extract and obtain cell components such as metabolites, proteins or nucleic acids. Thus, the effect of pH on the kinetics of mycelial growth of *I. obliquus* in a liquid medium at 28 °C was investigated. A rapid increase in the biomass was detected within the first six days, concurrent with the cells being in the exponential growth phase with significant consumption of carbon. On day eight, the mycelial biomass reached the apex, and the optimal growth of *I. obliquus* was observed at pH 6.2 with 0.18 g/100 mL of mycelial dry weight compared to 0.10 g/100 mL at pH 5 and 0.14 g/100 mL at pH 7.5 (Figure 3).

By-products of metabolism are shuttled throughout the cell and utilized to grow. Thus, optimal growth may influence the production of terpenoids [46]. In this study, different culturing conditions were used to establish the best possible parameters for *I. obliquus* growth. According to the results, the best condition for *I. obliquus* growth is at pH 6.2 and at 28 °C in a liquid culture that continuously shaken at 150 rpm. These results are supported by other studies on *I. obliquus* reporting similar optimal growth conditions [46,50,51]. The results of growth in the liquid and solid culture medium support that pH 6.2 would be the optimal pH for optimal mycelium growth.

For subsequent analyses, an *I. obliquus* liquid culture grown at 28 °C and pH 6.2 were used, since they showed optimal growth and morphological properties. In addition, it provided an easier system for the collection of cells for RNA extraction.

### 2.2. Illumina Sequencing and de Novo Assembly

To better understand the molecular mechanisms underlying the differences in chaga’s transcriptome caused by the presence of terpenoid substrates, RNA-Seq was performed. RNA was extracted from three biological replicates of cultured *I. obliquus* in three different conditions: In the absence (control, *n* = 3) or presence of the terpenoid substrate betulin (BET, *n* = 3) or white birch bark (WBB, *n* = 4). Ten corresponding cDNA libraries were generated and sequenced using Illumina HiSeq 4000 PE100. The raw reads for each library were deposited to the NCBI Sequence Read Archive under the accession PRJNA526077 (Appendix A—Table A1). A total of 229,970,255 raw paired reads were generated from all replicates of the three conditions of culturing. After filtering out low-quality sequences, 219,288,500 clean reads were obtained, corresponding to approximately 95% of the total raw reads (Appendix A—Table A1). Because of the lack of availability of information (i.e., genome, transcriptome) on *I. obliquus* or its related species, we combined all RNA-Seq libraries to build a deep transcriptome using de novo assembly. All clean reads obtained from the ten libraries were subsequently de novo assembled using the Trinity program (version 2.6.5), and a total of 196,273 transcripts with an average length of 2521 bp, and an N50 length of 4052 bp were obtained (Appendix A—Table A1). An evaluation of the size distribution showed that 96% of all transcripts of *I. obliquus* have lengths longer than 1 kb (Appendix A—Figure A2).

In a previous study, Zou et al. (2016) reported on the Illumina sequencing of *Inonotus baumii* [52] and obtained a total of 27,259,264 reads. After the Trinity de novo assembly, 30,051 unigenes with an average length of 561 bp and an N50 length of 831 bp were generated, which is two-to-three times less than our results [52]. In another research work on the saprophytic fungus *Wolfiporia cocos*, the Illumina sequencing of the transcriptome yielded a total of 38,722,186 reads, which were assembled into 60,354 contigs with an N50 of 765 bp [53]. In comparison to this, we obtained eight-to-ten times more reads, ensuring more coverage and allowing for a more contiguous assembly, as confirmed by the average transcript length of 2521 bp obtained here (Appendix A—Table A1).

The average transcript lengths of eukaryotic genes were greater than 1 kb. For example, the average transcript lengths of eukaryotic genes range from 1108 to 2667 bp in human and from 1135 to 1695 bp in yeast [54]. Additionally, eukaryotic proteins have an average size of 472 amino acid residues (i.e., 1419 bp), although the size of proteins from plant genomes are smaller than those of fungi and animals [55]. Based on our sequencing results, it could be concluded that the quality and the depth of this transcriptomic assembly is significantly improved compared to other fungal transcriptome studies.

### 2.3. Functional Annotation of the RNA-Seq Data

After the Basic Local Alignment Serach Tool (BLAST) annotation of the de novo assembly against the uniprot_sprot.trinotate_v2.0.pep protein database, a total of 20,072 transcripts with an average length of 4502 bp and an N50 length of 5512 bp were obtained (Appendix A—Table A1). A large number of transcripts had a similarity with known genes, suggesting a large amount of sequences specific to *I. obliquus*.

Gene function was annotated based on the following databases: Gene Ontology (GO), Clusters of Orthologous Groups of proteins (COG), and Protein family database (Pfam). A total of 86,246 transcripts (43.94% of assembled transcripts) had a match in the GO database with an E-value of 0 (Table 1). Additionally, 52,224 transcripts (20.05% of assembled transcripts) and 38,415 transcripts (19.57%) showed similarity to sequences in the COG and Pfam databases, respectively (Table 1).

**Figure 4:**
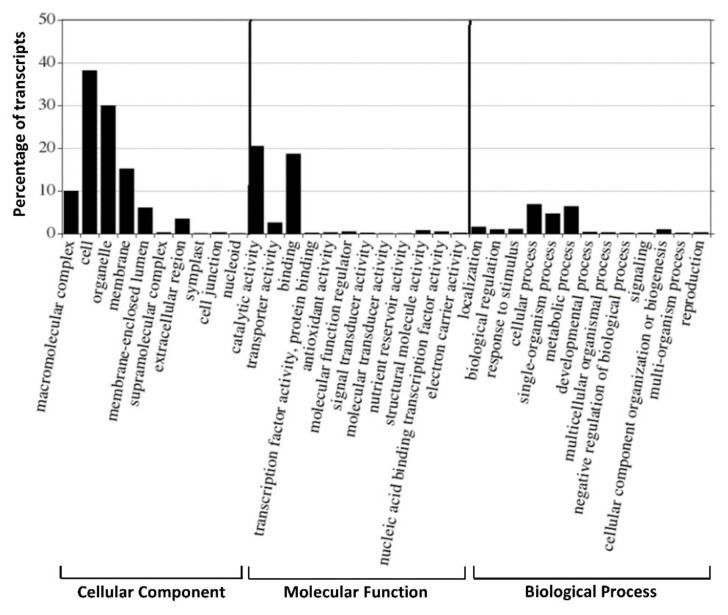
Gene Ontology (GO) terms of 41 functional groups of expressed transcripts from *Inonotus obliquus*.

The functional GO classification of genes is considered to be an effective tool, offering controlled vocabulary and strictly defined biological process for annotating and performing functional analysis of a large number of annotated genes and their products in a selected organism [56]. The GO classification was performed on the 196,273 transcripts, and a total of 86,246 transcripts were classified into 41 functional groups, categorized into three main GO ontologies: Biological process, cellular component and molecular function. The results indicated that the most highly annotated GO category was the cellular component (49.63%) with cell, organelle and membrane being the most abundant, while only a few transcripts were attributed to symplast, cell junction and nucleoid (Figure 4). Under the category of molecular function (38.42%), catalytic activity and binding activity were, respectively, the largest categories. Regarding biological process (11.95%), the dominant subcategories included genes involved metabolic process and cellular process (Figure 4). In addition, important categories with low numbers of transcripts were also represented in the GO, such as transcription factor activity, protein binding and nucleic acid binding.

**Figure 5:**
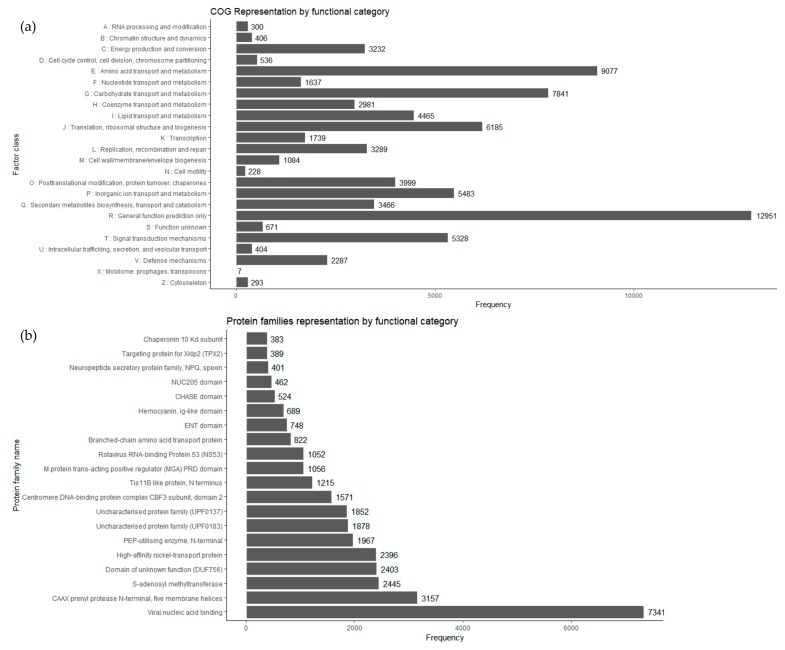
Annotation of the *Inonotus obliquus* transcriptome. (**a**) Cluster of orthologous groups (COG). (**b**) Protein family database (Pfam) classification of transcripts from *Inonotus obliquus.*

COG is a database where gene products from a common family ancestor are classified. From the *I. obliquus* transcriptome, COG-annotated putative proteins were functionally classified into, at least, 25 protein families involved in basic function, such as transcription, translation, signal transduction, cellular structure, biochemistry, metabolism, and molecular processing. The COG analysis of *I. obliquus* transcriptome led to the classification of 52,224 transcripts to COG classifications. The top COG categories included basic function prediction (16.63%), followed by amino acid transport and metabolism (11.65%), carbohydrate transport and metabolism (10.06%), translation, ribosomal structure and biogenesis (7.94%), inorganic ion transport and metabolism (7.03%), signal transduction, mechanisms (6.84%) and posttranslational modification, chaperones (5.13%) (Figure 5a). It is interesting that 4.45% of the *I. obliquus* annotated transcripts were ranked in the specialized metabolites biosynthesis, transport and catabolism, which suggests that specialized metabolism occurs in cultivated *I. obliquus* cells.

Protein domain analyses were performed using the Pfam database [57]. A total of 38,415 transcripts of *I. obliquus* were found to be associated with protein domains (Figure 5b). Compared to the *I. baumi* transcriptome, in which a total of 8276 transcripts contain at least one Pfam protein domain [52], our *I. obliquus* Pfam analysis was six times superior. This result provides another indication that our transcriptome was of good quality and had more depth.

Among the abundant protein families expressed, several were linked to primary metabolism enzymatic activities such as prenylprotease, *S*-adenosyl methyltransferase and phosphotransferase (PEP-utilizing enzyme). In addition, an important number of the expressed genes encoded predicted proteins from families implicated in ion transport, more specifically Nickel transport protein, a protein family proven to be essential for energy and nitrogen metabolism [58].

### 2.4. Differential Expression Analysis

**Figure 6:**
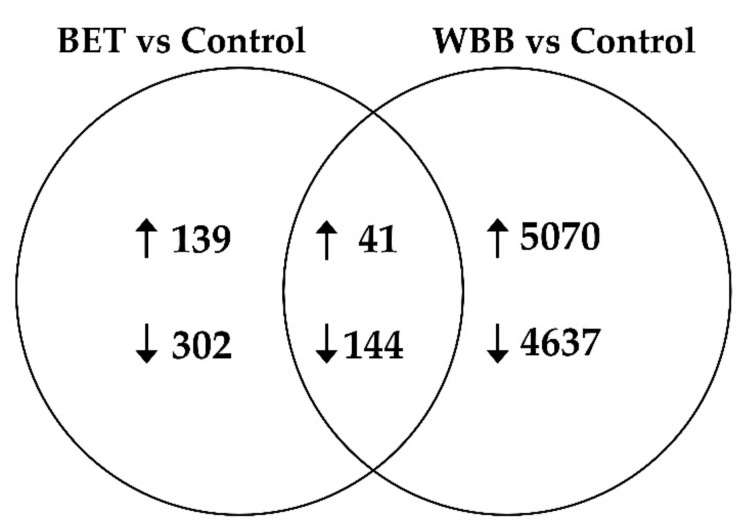
Venn diagram summarization of the differential expression comparisons. The number of differentially expressed genes (DEGs) in each circle represents the amount of DEGs between the different comparisons betulin (BET) versus control and white birch bark (WBB) versus control. Only the annotated genes were included. The overlapping number specifies the mutual DEGs between the distinctive comparisons and the non-overlapping numbers define the genes exclusive to each condition. Indicated in the diagram are the numbers of up-regulated (↑) and down-regulated DEGs (↓).

Differential expression analyses were performed between databases of *I. obliquus*. Transcripts with adjusted *p*-values ≤0.05 and a fold change (log2FC) ≥1 were designated as significantly differentially expressed transcripts. For *I. obliquus* cells cultivated with betulin (control vs. BET), the results showed that 441 differentially expressed transcripts (139 up-regulated and 302 down-regulated) were identified. Twenty-two times more transcripts (9707) were differently expressed in cells cultivated with bark (control vs. WBB), with a total of 5070 annotated transcripts up-regulated and 4637 transcripts down-regulated (Figure 6). This suggest that the birch bark has very different molecular impacts and requires more transcriptome adjustment from *I. obliquus* than betulin.

The top twenty-five up- and down-regulated expressed transcripts in *I. obliquus* cells cultivated with betulin (Appendix A—Table A2 and Table A3) or white birch bark (Appendix A—Table A4 and Table A5) were identified. After blasting the top twenty-five up- and down-regulated transcripts against the NCBI database, 48% could not be annotated (no hit). Among top-regulated transcripts with predicted function, 16% have an E-value of 10^−5^ or less (Appendix A—Table A2, Table A3, Table A4 and Table A5). Interestingly, the transcript annotated as phosphatidylinositol 4-phosphate 3-kinase ranked first up-regulated for both treatments, suggesting the importance of the signaling pathways involved in cell proliferation, cell survival, and intracellular protein trafficking (Appendix A—Table A2 and Table A4). Phosphatidylinositol 4-phosphate 3-kinase is a conserved enzyme involved in the regulation of phosphatidylinositol 4-phosphate, which is crucial for maintaining morphology, regulating lipid storage, Golgi function and actin cytoskeleton organization [59]. The signaling cascade activated downstream of this enzyme implicates the mitogen-activated protein kinase (MAPK) pathway, which regulates the filamentous growth of yeast [60]. This suggests a putative role for phosphatidylinositol 4-phosphate 3-kinase in the regulation of the morphology of the mycelium and filamentous growth in *I. obliquus* cells in presence of WBB or betulin.

In addition, caffeic acid 3-O methyltransferase, which catalyzes the conversion of caffeic acid to ferulic acid, was up-regulated in both treatments (Appendix A—Table A2 and Table A4). Phenolic acids, such as caffeic acid and ferulic acid, possess antioxidant properties that play key roles in the synthesis of polyphenols and protection against UV and oxidative stress [6,61,62,63]. The up-regulated condition of caffeic acid 3-*O*-methyltransferase in our transcriptomic results suggests an important role for ferulic acid in cultivated *I. obliquus* cells for protection or polyphenol synthesis.

The most down-regulated transcripts found in the betulin-cultivated cells were annotated as transcripts involved in cellular processes such as DNA replication, transcription, cell division and proliferation, i.e., t-RNA ligase, cyclin-dependent kinase, ribonucleoprotein complex subunits, chromosome proteins, and condensin complex subunits. This suggests betulin-induced cellular processes in *I. obliquus* cells (Appendix A—Table A3).

For WBB-cultivated cells (Appendix A—Table A5), most down-regulated transcripts found annotated for protein involved in metabolic processes such as several peroxidases, lipases, and racemases. This suggests that cultivation with WBB reduced the expression of genes encoding proteins involved in ligninolysis, i.e., to metabolize substrates/degrade bark molecules [64]. Effective lignin and plant cell wall degradation is possible through the action of enzymes from filamentous fungi, and it has been reported that fungi of the basidiomycetes family express several enzymes of the peroxidase family in their transcriptomes, such as lignin- and manganese-peroxidases known for their ability to degrade plant cell walls [65]. The down-regulated peroxidase transcripts in *I. obliquus* cells cultivated with WBB suggests that WBB may leak repressors for peroxidases to prevent its degradation from the mycelium.

### 2.5. Genes Involved in the Biosynthesis of Terpenoids in Cultured I. Obliquus Cells

The presence and identification of terpenoids in *I. obliquus* cells have been already studied and are well documented [1,22,28,35,40,41,43,46,66,67]. In this study, narrow BLAST searches were achieved to identify distinct transcripts encoding enzymes presumably taking part in terpenoid biosynthesis (Appendix A—Figure A1). Eighteen transcript sequences from the mevalonate pathway involved in terpenoid biosynthesis were found in the transcriptomic data of *I. obliquus* (Table 2). As expected, no genes involved in the non-mevalonate pathways were identified, suggesting the absence of this pathway in chaga. Similarly, the transcriptome from *I. baumii* reported no genes from the non-mevalonate pathway [52].

From the precursor pathway leading to IPP/DMAPP (Appendix A—Figure A1), several transcript variants of orthologous genes were identified (Table 2). For example, *AACT* genes have been cloned and characterized from various species including zebra fish, frog and human. Two human (*Homo sapiens* (Hs)) isoforms (HsAACT1 and HsAACT2) were identified to be ubiquitous and important enzymes found in different intracellular locations. The cytosolic HsAACT1 catalyzes the formation of the acetoacetyl-CoA required for sterol, including cholesterol biosynthesis. The exact function of HsAACT2 is not known, but patients with HsAACT2 deficiency have shown severe mental retardation and hypotonus [68]. Similarly, the model plant *Arabidopsis thaliana* (At) has two isoforms of AACT, where *AtAACT1* is primarily expressed in the vascular system and *AtAACT2* is deeply present in root tips, top stems, young leaves, and anthers. The characterization of T-DNA insertion of mutated alleles for each *AtAACT* locus established that *AtAACT2* function is necessary for normal male gamete transmission and embryogenesis, whereas plants lacking *AtAACT1* are viable with no apparent growth phenotype [69]. In yeast, *AACT* (also called *erg10*) encodes a multimeric enzyme, but the exact subunit structure has not been defined [68].

Transcripts encoding each enzyme involved in the formation of terpenoid precursors IPP and DMAPP (Appendix A—Figure A1) were identified in the transcriptome of *I. obliquus* (Table 2). According to the number of reads, the *HMGS, HMGR,* and *PDM* transcripts were the most abundant with E values of 0, suggesting that these gene sequences are well conserved.

Sesquiterpene synthases play an essential role in diversifying the skeletal structure of sesquiterpenoids by catalyzing the very complex cyclisation of the common precursor, FPP [38,70,71,72]. Sesquiterpenoids have been reported in Inonotus [43]; however, no biosynthetic genes have been identified. BLASTx searches of *I. obliquus* transcriptome led to the identification of three sesquiterpene synthases: Two transcript variants of the muurolene synthase (*MUS1* and *MUS2*) and one of the protoilludene synthase (*PRS*) (Table 2). *MUS1* and *MUS2* are only 36% identical at the nucleotide level, suggesting different functions, i.e., substrates for enzymatic activities. Similarly, the E values of *MUS1*, *MUS2* and particularly *PRS* suggest that their function may be different than orthologous sequences, suggesting that *I. obliquus* possess the machinery to produce structurally different sesquiterpenoids. Muurolene, protoilludene, and derivatives, also considered volatile organic compounds (VOCs), are produced by several fungal species. The rich bouquet of several VOCs contribute to the aroma and flavor of mushrooms. Though the ecological function of fungal volatiles is unknown, studies showed their biological activities as antibiotics, antioxidants or inhibitor of germination [72,73,74].

Lee et al. (2016) performed a transcriptomic analysis of the white rot fungus *Polyporus brumalis* which led to the identification of two transcripts, germacrene A synthase and trichodiene synthase, involved in sesquiterpenoid biosynthesis [75]. However, the final products of these enzymes were not detected in the cultivated fungus [75]. To our knowledge, this study is the first to report the presence of transcripts encoding sesquiterpenoid-forming enzymes in Inonotus.

Triterpenoids from Inonotus species include high pharmaceutical valued metabolites. For example, the lanosterol-derived triterpenoid inotodiol and the lupeol derivative betulinic acid are well known bioactive triterpenoids from *I. obliquus* [17,19,24,25,27,28,33,36,76,77]. Though several studies have shown the presence of triterpenoids in *I. obliquus*, only one gene, SQS, has been identified [78]. In order to increase knowledge on genes involved in triterpenoids production, a search for these genes was carried out in the transcriptome of *I. obliquus*. Two transcript variants of squalene synthase (*SQS1* and *SQS2*) were identified with a 95% sequence identity to each other (*SQS2* has an insertion at the 3′ end) (Table 2). Our *SQS1* transcript sequence is identical to previously characterized *SQS* from *I. obliquus* [78].

A single transcript for *AO* was identified in the transcriptome of *I. obliquus*, whereas two transcript variants, *LAS1* and *LAS2*, were found (Table 2). *LAS1* and *LAS2* possess a 98% sequence identity, but *LAS2* is longer and far less expressed than *LAS1*, suggesting the importance of *LAS1* in the production of lanosterol and its derivatives in *I. obliquus* cell cultures (Table 2).

According to the literature, only one terpenoid gene, *SQS*, has been characterized from *I. obliquus,* whereas nine genes related to terpenoids biosynthesis where identified in *I. baumii* [52,78]. The transcriptome generated in the current study allowed for the identification of all (17) orthologous genes involved in terpenoid metabolism. Furthermore, variants of the *AACT*, *MUS*, *SQS*, and *LAS* genes were identified, indicating that these steps may be regulated differently in *I. obliquus*. For example, the higher expression of *AACT1*, *MUS1*, and *LAS1* suggests the importance of theses isoforms in terpenoid formation of *I. obliquus*-cultivated cells.

**Figure 7:**
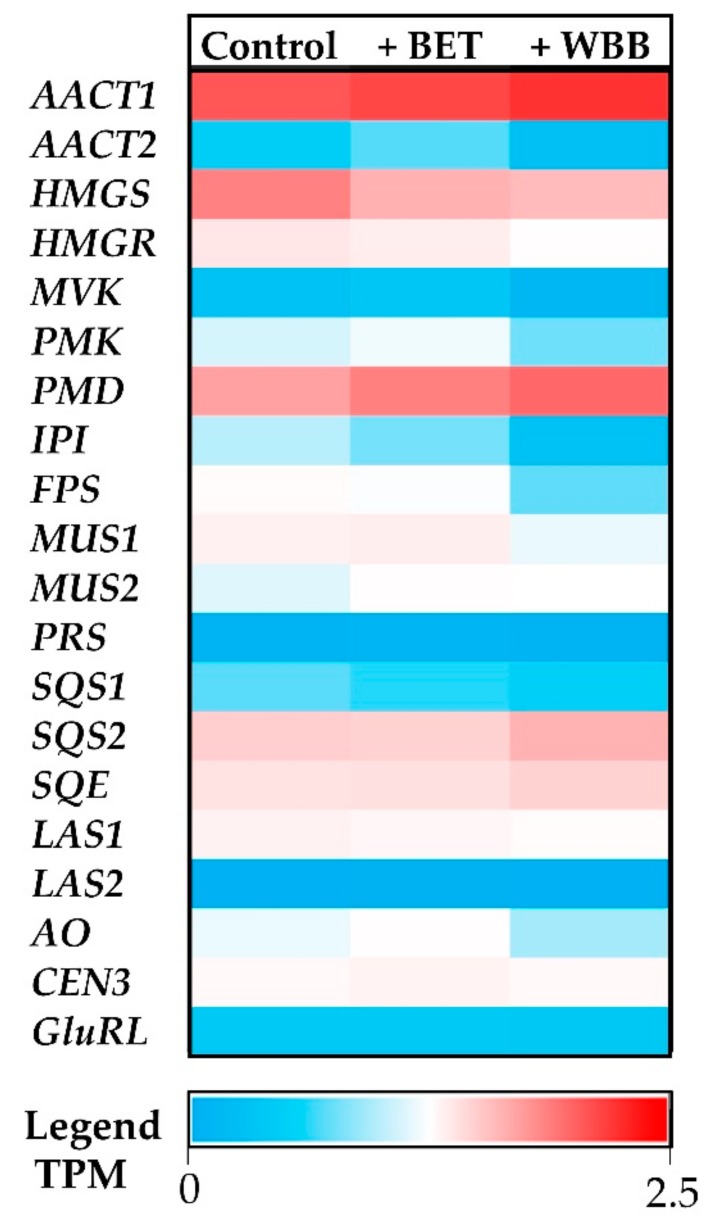
Heatmap of the digital expression levels of transcripts encoding terpenoid biosynthetic enzymes in the *Inonotus obliquus* transcriptome. Results are in transcripts per million (TPM) with the legend from low (blue) to high (red) expressed transcript.

**Figure 8:**
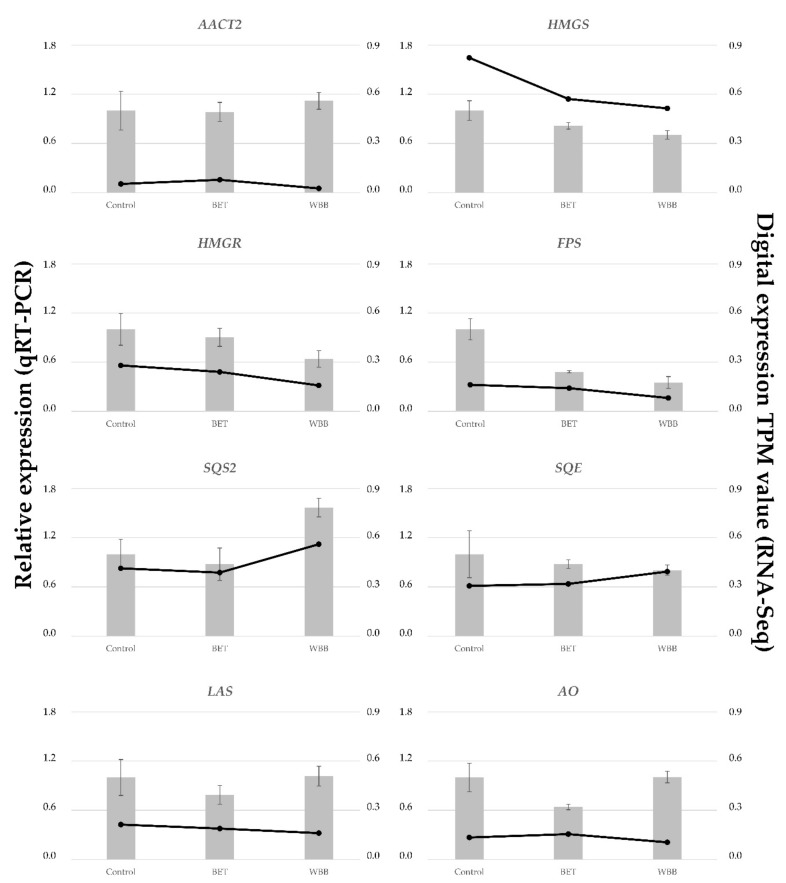
Comparison of expression profiles of eight representative transcripts from *I. obliquus* cells supplemented without (control) or with betulin (BET) or white birch bark (WBB), as measured by RNA-Seq and quantitative reverse transcription PCR (qRT-PCR). The eight transcripts are assigned to the terpenoid pathway in Appendix A—Figure A1. Columns represent expression determined by qRT-PCR (left *y*-axis), while lines represent digital expression by RNA-Seq in TPM values (right *y*-axis). The *x*-axis indicates different growth conditions (control, BET, and WBB). Graphs are plotted using normalized ddCt values scaled to control. *Centromere protein 3* (CEN3) was used for internal reference. Expression fold change and error bars were calculated using the comparative 2^−ΔΔCt^ method [84]. Bars represent the mean standard deviation of three independent replicates. Abbreviations are defined in Appendix A—Figure A1.

The digital expression of each terpenoid-associated transcript was studied for each growth condition (Figure 7). Upstream precursor genes, such as *AACT1* and *PMD*, had a higher expression in *I. obliquus* cells compared to specific downstream (sesqui and triterpenoid) genes such as *PRS* and *LAS2* (Figure 8). From the precursor pathway, the most strongly expressed transcript, *AACT1*, remained highly expressed across the different treatments, suggesting no transcriptional regulation under those conditions, whereas *PMD*, *SQS2* and *SQE* were up-regulated in presence of BET and WBB (Figure 7). In contrast, *HMGR*, *MUS1*, and *AO* were down-regulated in presence of the terpenoid substrate, particularly in *I. obliquus* cells cultivated with WBB. HMGR is a rate-limiting enzyme in the terpenoid biosynthesis. It is possible that the addition of terpenoid substrates promotes negative feedback regulation mechanisms. For example, HMGR, which generates mevalonate, a critical intermediate in the biosynthesis of terpenoids, is exposed to large amount of feedback regulation through various mechanisms that are influenced by end-products, light, and hormones [79,80,81,82].

### 2.6. qRT-PCR Validation of RNA-Seq Gene Expression Data

To validate the RNA-Seq digital expression data, eight transcripts encoding terpenoid biosynthetic enzymes were selected for the qRT-PCR analysis. For control reference genes, transcripts with no/low variation were extracted from the database using a custom method developed by dos Santos et al. (2019), which has shown to outperform pre-defined references genes [83]. Indeed, *centromere protein 3* (*CEN3*) and *glutamine RNA ligase* (*GluRL*) show no/low difference of expression among the conditions tested indicating that they are good reference genes for qRT-PCR study (Figure 7).

All eight transcripts qRT-PCR analyses are consistent with RNA-Seq results (Figure 8). This indicates that our transcriptome was reliable and that we could make reasonable inferences from the differentially expressed transcripts. For example, as observed for the digital expression, *SQS2* was up-regulated and *HMGR* was down-regulated in presence of terpenoid substrates ( Figure 7 and Figure 8).

Interestingly, qRT-PCR analysis showed a downregulation of *FPS* in the presence of terpenoid substrates, suggesting a negative feedback regulation on this important step in the sesqui and triterpenoid biosynthesis. In contrast, *SQS2* expression increased in the presence of WBB, suggesting a positive regulation. This opposite regulation, negative on *FPS* and positive on *SQS2*, may be due to the presence of terpenoids such as FPP in bark residues. Altogether, the results suggest that few key reactions in the terpenoid pathway are regulated at the transcriptional level in presence of terpenoid substrates in *I. obliquus* cell cultures.

The amounts of valuable terpenoids accumulated remained low under exogenous induction in a fungal culture. The metabolic engineering of microorganisms is an interesting alternate route for the production of these important compounds. To do this, a better understanding of the terpenoid biosynthetic pathway and the genes involved in this pathway is required. However, the lack of genomic information about *I. obliquus* hinders the development of alternative production methods. In this work, the best conditions for growth of *I. obliquus* cells cultivated in presence of terpenoid substrates, betulin or white birch bark were examined, and the corresponding transcriptomes obtained after next-generation sequencing and de novo assembly. Transcriptome analysis identified eighteen transcripts encoding enzymes implicated in the terpenoid metabolism. Comparative analyses of the transcriptomes yielded valuable information with respect to the wide variety of genes implicated in terpenoid metabolism in *I. obliquus* cells under the described conditions. It would be interesting to compare the laboratory-adapted isolate of *I. obliquus* to the wild type fungus to monitor gene expression implicated in the host-pathogen interaction between chaga and its host, birch trees.

## 3. Materials and Methods

### 3.1. Chemicals and Reagents

Standard betulinic acid (97%) was purchased from Adipogen Corp. (San Diego, CA, USA). Standard betulin (98%) was purchased from Sigma-Aldrich (Saint-Louis, MO, USA). Methanol, acetonitrile and dimethylsulphoxide (DMSO), ethyl acetate, and acetic acid were HPLC grade from Fisher Chemical. The other cited chemicals were of analytical grade. Media components (yeast extract, malt extract, agar, etc.) were purchased from Sigma-Aldrich (Saint-Louis, MO, USA).

### 3.2. Fungal Culture and Growth Conditions

*I. obliquus* was provided and cultured by the Biopterre laboratory (La Pocatière, Qc, Canada). The stock of *I. obliquus* was maintained on a potato dextrose agar (PDA) medium, and the stock culture was stored at 4 °C. The identity of the isolate was confirmed by PCR amplification followed by Sanger sequencing of the ribosomal internal transcribed spacer (ITS) DNA region. The sequence data were deposited to the NCBI database under the GenBank accession number MN239482. To optimize the mycelial growth of *I. obliquus* in laboratory, yeast malt broth (YMB) and yeast malt agar (YMA) media were selected based on previous reported studies [51,67,85]. The YMB and YMA contained 3 g/L of yeast extract and 3 g/L of malt extract. Different growth condition parameters of the liquid and solid culture of *I. obliquus*, were tested: Three pH (5, 6.2, and 7.5) and three temperatures (22 °C, 28 °C, and 37 °C) in presence or not of white birch bark (50 µg/mL) or betulin (15 µg/mL).

#### 3.2.1. Optimization of Growth Conditions for Solid Medium

To optimize the mycelial culture on solid media, the effect of pH, temperature, and the presence of white birch bark (50 µg/mL) were investigated. *I. obliquus* was initially grown for 14 days on a YMA medium in petri dish in absence of light. A disc of 1 cm^2^ was cut after 14 days of culture, and then it was transferred into other plate under different conditions for 14 days in absence of light. The growth diameter was measured, in triplicate, every two days on the Petri dish [86,87]. In order to sustain the culture of *I. obliquus,* mycelium was maintained on YMA Petri dishes for three months in an incubator at 28 °C and then stored at 4 °C. Based on preliminary results obtained from the optimization of growth in a solid culture, and in order to reduce the parameters in liquid cultures, the optimization of the liquid cultures of *I. obliquus* was carried out at 28 °C, which yielded the best growth of the isolate.

#### 3.2.2. Optimization of Growth Conditions for Liquid Medium

To initiate a liquid culture of *I. obliquus*, a one-centimeter^2^ disc was collected from a fresh solid culture of mycelium in a YMA medium. The mycelium pre-culture was grown in flask of 250 mL with 30 mL of yeast malt broth (YMB) at 28 °C on a rotary shaker incubator at 150 rpm for 8 days. Then, to investigate the effect of pH (5, 6.2 and 7.5), an aliquot of 10 mL of the mycelium was transferred to 250 mL flask containing 100 mL of YMB at different pH, incubated at 28 °C on a rotary shaker (Thermofisher Scientific) at 150 rpm in absence of light for 8 days. The mycelium was sampled every 48 h by sequential filtration, washed several times with distilled water, and then was dried overnight at 70 °C.

### 3.3. Isolation of RNA

Ten mL aliquote of 8 days *I. obliquus* liquid pre-culture were aseptically added to 250 mL flask containing 100 mL of a YMB medium supplemented or not with 0.2% DMSO (Control), 15 µg/mL of betulin (BET), or 50 µg/mL of white birch bark (WBB), and grown for 8 days on a rotary incubator at 150 rpm and 28 °C in absence of light. The *I. obliquus* mycelium was collected after 8 days of growth. The mycelium was filtered, frozen with liquid N_2_, and then placed in a 2 mL tube with 2 mL of TRIzol reagent (Fisher Scientific, Toronto, Canada). The mycelia were crushed using the TissueLyser II (QIAGEN, Qiagen Retsch GmbH, Hannover, Germany) using the stainless steel beads with 5 mm diameter (QIAGEN, Qiagen Retsch GmbH, Hannover, Germany) at speed of 30 strokes per second for 3 min until a homogeneous sample was obtained. The tubes were incubated on ice for 5 min, and then 400 µL of chloroform were added. The tubes were vigorously vortexed for 15 s and incubated in an ice bath for 3 min. After centrifugation at 13,000× *g* for 15 min at 4 °C, two phases were obtained, which were separated by cellular debris—the clear phase of the supernatant was recovered and transferred into another clean 1.5 mL Eppendorf tube. RNA was precipitated with the addition of 700 μL isopropanol, followed by incubation at −20 °C for 3 h. Finally, precipitated RNA was collected by centrifugation at 13,000× *g* for 15 min at 4 °C, and the pellets were washed once with 1 mL of ice cold 75% ethanol and air dried briefly at room temperature. Then, they were centrifuged again at 13,000× *g* for 5 min at 4 °C. The RNA pellets were resuspended using 20 μL of nuclease free water. The quality and quantity of RNA extracted from the samples under different conditions were verified using the Nanodrop and the Bioanalyzer. Only samples with an RNA Integrity number higher than 8 were selected for Illumina sequencing.

### 3.4. Transcriptome Sequencing, de Novo Assembly and Functional Annotation

Sequencing was performed using Illumina HiSeq 4000, PE 100 paired ends, at McGill University and Genome Québec Innovation Centre (Montreal, QC, Canada) on cDNA Libraries converted from isolated high-quality mRNA. Among the raw reads obtained from Illumina sequencing, low quality reads, reads with unknown nucleotides, and reads with adapters were found (Table 3). Trimmomatic was used to remove the adapters from sequencing and trimmed reads from the 3′ end and further filtered all reads below 50 bp in order to obtain clean reads [88]. However, once the data from the surviving pairs were generated, Trinity normalization was performed to remove redundant reads in datasets without affecting its *k*-mer content [89]. De novo assembly of cleaned and standardized reads was performed using Trinity (v2.6.5) assembler [90]. Trinity method assembles the RNA-Seq reads into full-length transcripts, which are called contigs or unigenes. All the unigenes were aligned to the public protein database uniprot_sprot.trinotate_v2.0.pep protein database using the BLASTX program against the NCBI BLAST families using an E-value of 10^−5^. Top BLAST hits were used for annotation of component/gene for each transcript. To quantify the gene transcript abundance, the raw RNA-Seq reads were mapped to assembled transcripts with Bowtie [91] using default parameters. The gene transcript abundance was calculated as transcripts per kilobase million (TPM) using the RSEM package [92].

Functional annotations of unigenes were performed using Trinotate (http://trinotate.github.io/), by aligning our transcripts to Swiss Institute of Bioinformatics databases (Swiss-Prot) with BLASTX and by identifying protein domains (HMMER/PFAM), protein signal peptides, transmembrane domains prediction (signalP/tmHMM), and Clusters of Orthologous Groups (COG). Then, the functional annotation of unigenes was compared to currently curated annotation databases (EMBL Uniprot eggNOG/GO Pathways databases). The Trinity assembly and functional annotation of unigenes were integrated as an annotation report into an SQLite database.

Gene Ontology (GO) annotations for our unigenes were obtained using the Swiss-Prot. The WEGO 2.0 software was used to obtain the GO functional classification for all unigenes and to better understand the distribution of gene functions in *I. obliquus* at the macro level. All annotated sequences were linked to the GO terms in the database, and a quantification of the number of sequences for each of the different terms was subsequently calculated. The raw reads under the accession number PRJNA526077 were deposited to the Sequence Read Archive [93].

### 3.5. qRT-PCR Analysis

Quantitative reverse transcription polymerase chain reaction (qRT-PCR) was performed on *I. obliquus* in different culture conditions (control, with white birch bark (WBB) or with betulin (BET). The qRT-PCR assay was performed using a CFX connect Real-Time System by BioRad and analyzed using BioRad CFX Maestro 1.1. (BioRad laboratories) including statistics analysis using an ANOVA.

Two micrograms of total RNA extracted from different culture conditions were reverse transcribed into single-stranded complementary DNA using the High-Capacity RNA-to-cDNA™ Kit supplied by Thermo Fisher Scientific, following their protocol. qRT-PCR was performed in triplicates in a 10 µL total volume reaction containing 6 µL of SYBR Lo-ROX mix from Bioline SensiFAST SYBR Lo-ROX mix kit (FrogaBio, Toronto, ON, Canada), 2 µL cDNA and 2 µL of 200 µM of specific primers. The primers were designed using the conserved sequences of the variant of the transcripts selected using Clustal tool on Galaxy platform. The primers were aligned to the target genes sequences using the Integrated DNA Technology (www.idtdna.com), Primer3 and Tm Calculator (New England Biolabs, tmcalculator.neb.com) to select suitable annealing temperatures for each primer. The qRT-PCR was performed for the following gene transcripts: *Acetoacetyl-CoA synthetase (AACS), hydroxymethylglutaryl-CoA synthase A (HMGS); 3-hydroxy-3-methylglutaryl-coenzyme A reductase (HMGR), farnesyl pyrophosphate synthase (FPS), squalene synthase (SQS), squalene monooxygenase (SQE), β-amyrin synthase (βAS)* and *lanosterol synthase (LAS).* The primers used for the qRT-PCR assay are listed in Table 4. qRT-PCR data were standardized using reference genes. From the transcriptomic data of *I. obliquus*, we selected as reference genes, the two genes with lowest variability in their expression under the different culture conditions. The Ct values for the seven genes of interests were normalized to the Ct value of the reference gene *centromere protein 3 (CEN3*) and *glutamine RNA ligase* (*GluRL*).

The amplification was carried out under the following conditions: 2 min of polymerase activation at 95 °C, followed by 39 cycles of 5 sec of denaturation at 95 °C, 10 sec of annealing, and 30 s of extension and fluorescence data acquisition at 72 °C were measured. After verifications of unique and clear melt curve and unique band obtained on agarose gel electrophoresis, RT-qPCR efficiency and linearity were taken into account for next step of qRT-PCR. The percentage of efficiency qRT-PCR was chosen between 90% and 110% and a standard curve correlation coefficient (R2) ≥96 [94].

### 3.6. Accession Numbers

The sequences described in this paper have been deposited in the National Center for Biotechnology Information Sequence Read Archive (https://www.ncbi.nlm.nih.gov/sra/) under the accession number PRJNA526077. Gene transcript sequences were deposited in Genbank with the following accession numbers for nucleotide sequences: Acetoacetyl-CoA transferase 1 (MK825552); acetoacetyl-CoA transferase 2 (MK825553); HMG-CoA synthase (MK825554); HMGR, HMG-CoA reductase; (MK825555); MVA kinase (MK825556); phosphoMVA kinase (MK825557); diphosphoMVA decarboxylase (MK82558); IPP isomerase (MK825559); FPP synthase (MK825560); Muurolene synthase 1 (MK825561); Muurolene synthase 2 (MK825562); Protoilludene synthase (MK825563); Squalene synthase 1 (MK825564); Squalene synthase 2 (MK825565); Squalene epoxidase (MK825566); Lanosterol synthase 1 (MK825567); Lanosterol synthase 2 (MK825568); 11-oxo-β-amyrin 30-oxidase (MK825569); Centromere protein 3 (MK825570); Glutamine RNA ligase (MK825571) and Glyceraldehyde 3-phosphate deshydrogenase (MK825572).

## 4. Conclusions

This research allowed us to define the best growth conditions to conduct qRT-PCR and RNA-Seq de novo assembly of Québec *Inonotus obliquus*. The best growing conditions in liquid and solid cultures were determined for optimal production yield of *Inonotus obliquus* mycelia. It was found that optimal conditions were obtained at pH 6.2, 28 °C, and using YMB as a culture medium. In this study, we provide the first transcriptomic report generated by next-generation sequencing (Illumina HiSeq4000) of the medicinal fungus chaga, *I. obliquus,* grown under different conditions. The assembled transcriptomes generated 196,273 transcripts. High-quality transcriptomes were obtained, and based on genes annotations, we were allowed to identify of transcripts encoding 18 enzymes playing a key role in the terpenoid pathway. This led us to identify novel gene sequences encoding biosynthetic enzymes involved in the terpenoid pathway in chaga. The transcriptomic analysis of this medicinal fungus has led to the discovery of new genes that can be used as a reference for future genetic and genomic studies on chaga and other related medicinal species. Moreover, this transcriptome assembly may also be used as a reference platform for studies on fungus Hymenochaetaceae family. Beyond the biological impact, the data can be used as a reference to provide gene sequences for metabolic engineering and should pave the way for advanced fungal biology and biotechnology to produce valuable chaga terpenoids.

## Figures and Tables

**Table 1:** Summary of functional annotation for *Inonotus obliquus* transcripts in public databases.

Description	Number of Transcripts	Percentage (%)
Blast-GO annotations	86,246	43.94
COG annotations	52,224	20.05
Pfam annotations	38,415	19.57
Total number of sequences not annotated	105,655	53.83
Total number of sequences annotated	90,618	46.17
Total of sequences transcripts	196,273	100.00

**Table 2:** Summary of the coding DNA sequence (CDS) of biosynthetic gene transcripts identified from the *I. obliquus* transcriptome known to be involved in terpenoid metabolism. All transcripts were deposited in the publicly available Genbank sequence database with the corresponding accession numbers listed in the material and methods section. * Accession number to UniprotKB/Swiss-Prot.

Name	Number of Reads (Control)	Length (nt)	CDS (nt)	Top Annotation	Species	E Value	* Accession Number
*AACT1*	8769	1655	816	Acetyl-CoA acetyltransferase	*Danio rerio*	1 × 10^−64^	Q6AZA0.1
*AACT2*	13	1247	1167	Acetyl-CoA acetyltransferase	*Xenopus laevis*	4 × 10^−149^	Q6GN02.1
*HMGS*	15,322	2277	1434	3-hydroxy-3-methylglutaryl CoA synthase	*Ustilago maydis*	0	Q4P3F1.1
*HMGR*	12,463	6041	2814	3-hydroxy-3-methylglutaryl CoA reductase	*Phycomyces blakesleeanus*	0	Q12649.2
*MVK*	323	1872	927	Mevalonate kinase	*Homo sapiens*	3 × 10^−39^	Q03426.1
*PMK*	4024	5489	1440	Phosphomevalonate kinase	*Arabidopsis thaliana*	2 × 10^−61^	Q9C6T1.1
*PMD*	10,182	4189	1209	Diphosphomevalonate decarboxylase	*Ganoderma lucidum*	0	G9BIY1.1
*IPI*	1668	1830	969	Isopentenyl diphosphate isomerase	*Xanthophyllomyces dendrorhous*	2 × 10^−106^	O42641.1
*FPS*	5469	4639	771	Farnesyl diphosphate synthase	*Schizosaccharomyces pombe*	7 × 10^−113^	O14230.1
*MUS1*	5999	4901	1251	Alpha-muurolene synthase	*Coprinopsis cinerea*	1 × 10^−104^	A8NE23.1
*MUS2*	2651	5374	1281	Alpha-muurolene synthase	*Coprinopsis cinerea*	7 × 10^−169^	A8NE23.1
*PRS*	55	2054	258	Delta (6)-protoilludene synthase	*Armillaria gallica*	1 × 10^−20^	P0DL13.1
*SQS1*	2007	3770	1476	Squalene synthase	*Ustilago maydis*	2 × 10^−154^	Q92459.2
*SQS2*	4675	2525	1332	Squalene synthase	*Ustilago maydis*	4 × 10^−147^	Q92459.2
*SQE*	9978	6694	1164	Squalene epoxidase	*Homo sapiens*	3 × 10^−88^	Q14534.3
*LAS1*	3491	2500	1695	Lanosterol synthase	*Pneumocystis carinii*	0	Q96WJ0.1
*LAS2*	5	2447	2020	Lanosterol synthase	*Pneumocystis carinii*	0	Q96WJ0.1
*AO*	4214	6834	1596	11-oxo-beta-amyrin 30-oxidase	*Glycyrrhiza uralensis*	2 × 10^−39^	H1A988.1
*CEN3*	1727	2261	1254	Centromere protein 3	*Schizosaccharomyces pombe*	9 × 10^−22^	Q9USR9.2
*GluRL*	21	1656	831	Glutamate tRNA ligase	*Pelodictyon luteolum*	2 × 10^−35^	Q3B256.1

**Table 3:** List of *Inonotus obliquus* RNA-Seq library sequenced using Illumina HiSeq4000 deposited to the NCBI Sequence Read Archive under the accession PRJNA526077.

Library	Nb of Reads	Nb of Bases
RNA-Seq of *Inonotus obliquus* cell culture: Control 1	41,874,533	8,374,906,600
RNA-Seq of *Inonotus obliquus* cell culture: Control 2	19,534,645	3,906,929,000
RNA-Seq of *Inonotus obliquus* cell culture: Control 3	22,008,711	4,401,742,200
RNA-Seq of *Inonotus obliquus* cell culture: Betulin1	25,264,465	5,052,893,000
RNA-Seq of *Inonotus obliquus* cell culture: Betulin2	20,205,852	4,041,170,400
RNA-Seq of *Inonotus obliquus* cell culture: Betulin3	22,305,852	4,461,046,200
RNA-Seq of *Inonotus obliquus* cell culture: White birch bark 1	17,766,031	3,553,206,200
RNA-Seq of *Inonotus obliquus* cell culture: White birch bark 2	16,318,641	3,263,728,200
RNA-Seq of *Inonotus obliquus* cell culture: White birch bark 3	28,913,696	5,872,739,200
RNA-Seq of *Inonotus obliquus* cell culture: White birch bark 4	15,777,829	3,155,565,800
Total	229,970,255	46,083,926,800

**Table 4:** Primers used for qRT-PCR validation of gene expression.

Gene	Forward Primer (5′-3′)	Reverse primer (5′-3′)	Product Size (bp)
*AACT2*	CCGATCACTGTGAAGGGTAAG	TGGTGCGATAGGGAAATCTATG	273
*HMGS*	CAGTGTCGACTACCCCGTTT	GTGTACATGTTTCCGCAACG	280
*HMGR*	GTCGTTCCTGGTGTGAGGTT	GCGTCTTAGTGGCCAGAGTC	243
*FPS*	TCATGCACGAAACGACTCTC	CCGAAGCAGTCGAGGTAGTC	276
*SQS2*	CTTCGAGGGTTGGACACAAT	GACGTCGCGGTAAGAAGAAG	282
*SQE*	GTCCTGTCCTACTCTACCAAATC	GTCATTCCACCTCCAGTCAA	289
*LAS*	GCCTGAAGGCTGTGCTTTAC	TCTGCAAGGAAAGCATTGTG	246
*AO*	GCTTACTCCTGCGTTCAGTAA	TCCGAAACCTCCTCCATAGT	375
*CEN3*	AGGTCGACCGAGAAGTCG	CTTGAACTTTCTTACGTTTG	300

**Figure A1:**
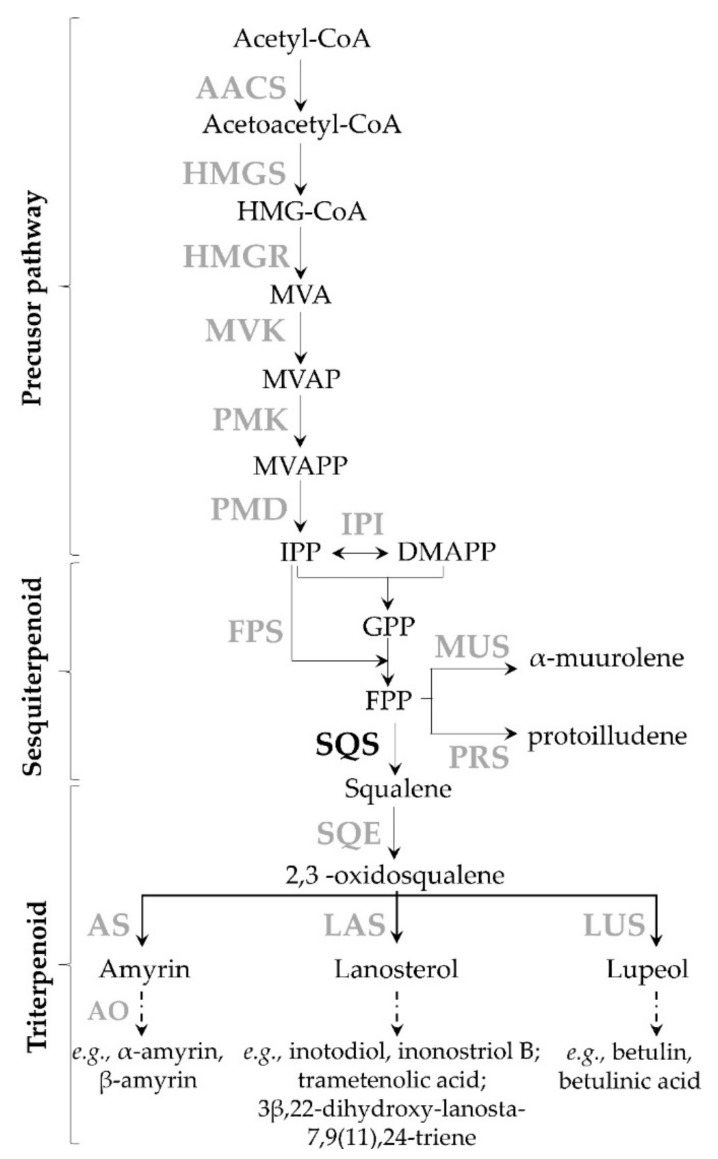
Proposed biosynthetic pathway leading to multiple terpenoids in *I. obliquus*. Enzymes for which corresponding genes have been isolated from *Inonotus* are shown in bold black whereas the ones from other species are shown in bold grey. Broken arrow represents more than one biochemical reaction. Abbreviations: HMG-CoA, 3-hydroxy-3-methylglutaryl-CoA; MVA, mevalonic acid; MVAP, MVA phosphate; MVAPP, MVA diphosphate; IPP, isopentenyl diphosphate; DMAPP, dimethylallyl diphosphate; GPP, geranyl diphosphate; FPP, farnesyl diphosphate; AACT, Acetoacetyl-CoA transferase; HMGS, HMG-CoA synthase; HMGR, HMG-CoA reductase; MVK, MVA kinase; PMK, phosphoMVA kinase; PMD, diphosphoMVA decarboxylase; IPI, IPP isomerase; FPS, FPP synthase; MUS, Muurolene synthase; PRS, Protoilludene synthase; SQS, Squalene synthase; SQE, Squalene epoxidase; AS, Amyrin synthase; βAO, 11-oxo-β-amyrin 30-oxidase; LAS, Lanosterol synthase; LUS, Lupeol synthase.

**Figure A2:**
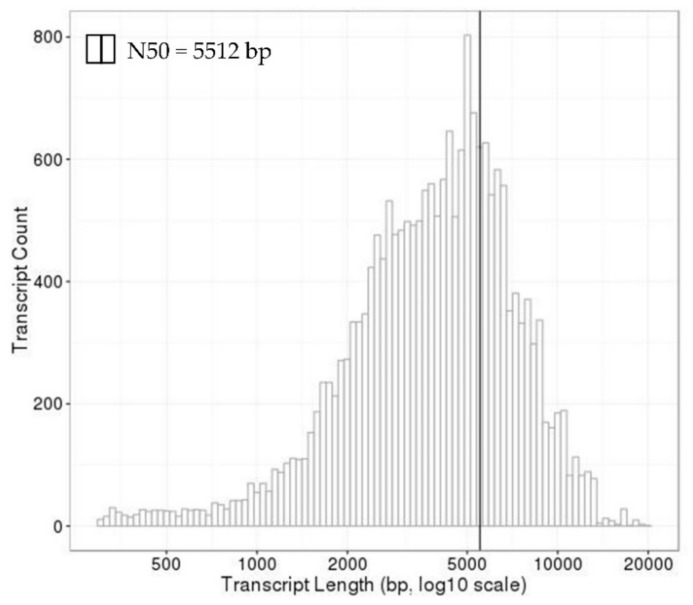
Sequence length distribution of transcripts of *I. obliquus* after Trinity de novo assembly.

## Abbreviations

YMA	Yeast malt agar
YMB	Yeast malt broth
WBB	White birch bark
HMG-CoA	3-hydroxy-3-methylglutaryl-CoA
HMGR	3-hydroxy-3-methylglutaryl-CoA reductase
MVA	mevalonic acid
MVAP	MVA phosphate
MVAPP	MVA diphosphate
IPP	isopentenyl diphosphate
DMAPP	dimethylallyl diphosphate
GPP	geranyl diphosphate
FPP	farnesyl diphosphate
AACT	Acetoacetyl-CoA transferase
HMGS	HMG-CoA synthase
HMGR	HMG-CoA reductase
MVK	MVA kinase
PMK	phosphoMVA kinase
PMD	diphosphoMVA decarboxylase
IPI	IPP isomerase
FPS	FPP synthase
MUS	Muurolene synthase
PRS	Protoilludene synthase
SQS	Squalene synthase
SQE	Squalene epoxidase
AS	Amyrin synthase
AO	11-oxo-β-amyrin 30-oxidase
LAS	Lanosterol synthase
LUS	Lupeol synthase

## Appendix A. Supplementary Information

In fungi, terpenoid biosynthesis occurs via the mevalonate (MVA) metabolic pathway [38,95,96,97] which can be divided into three parts (Appendix A—Figure A1). The first part (precursor pathway) involves a series of enzymatic reactions converting acetyl-CoA to isopentenyl diphosphate (IPP) and dimethylallyl diphosphate (DMAPP) which are the precursors of all terpenoids. For the second part (sesquiterpenoid pathway), the cytosolic farnesyl diphosphate synthase (FPS) condenses two molecules of IPP with one molecule DMAPP head-to-tail sequentially to produce farnesyl diphosphate (FPP). FFP serves as a precursor for sesquiterpenoids, which are synthesized by terpene synthases and can be decorated by other various enzymes. For example, the cyclization of FPP by characterized fungal sesquiterpenoid synthases such as muurolene synthase (MUS) or protoilludene synthase (PRS) followed by subsequent reactions yield diverse sesquiterpenoids. MUS catalyzes the 1,10 cyclization of FPP to α-muurolene and germacrene A, and six corresponding genes (*Cop1–6*) were identified through mining of the genome of *Coprinus cinerea* [70,71,98]. Lastly, the third part of the pathway involves reactions catalyzing the formation of three groups of triterpenoids: The amyrin-type, the lanosterol-type, and the lupeol-type. Briefly, at the endoplasmic reticulum, squalene synthase (SQS) catalyzes the condensation of two FPP molecules to produce squalene, which is the first committed precursor for triterpenoids and steroids biosynthesis. Squalene epoxidase (SQE) catalyzes the oxide squalene formation, which is modified by various tailoring enzymes. For example, lanosterol is formed from oxide squalene by a cyclization reaction catalyzed by lanosterol synthase (LAS) and is further metabolized into triterpenoids such as inotodiol [52,99]. To date, only the *SQS* sequence has been identified, cloned and characterized from *I. obliquus* (Appendix A—Figure A1) [78].

**Table A1:** Results of Illumina sequencing output and assembly for *Inonotus obliquus*.

Read Trimming and Clipping of Adapters	
Raw paired reads ^a^	229,970,255
Surviving paired reads ^b^	219,288,500
Surviving paired reads (%) ^c^	95.36
**Trinity de novo Assembly**	
Nb. Transcripts ^d^	196,273
Nb. Components ^d^	42,787
Total Transcripts Length (bp)	494,954,509
Max. Transcript Length (bp)	2338
Min. Transcript Length (bp)	201
Median Transcript Length (bp)	1953
Mean Transcript Length (bp)	2521
N50 (bp) ^e^	4052
GC percentage (%)	50.07
**BLAST annotation and filtered annotated components**	
Nb. Transcripts	20,072
Nb. Components	2224
Total Transcripts Length (bp)	90,372,815
Max. Transcript Length (bp)	19,856
Min. Transcript Length (bp)	299
Median Transcript Length (bp)	4074
Mean Transcript Length (bp),	4502
N50 (bp) ^f^	5512
GC percentage (%)	49.50

^a^ Number of paired reads obtained from the sequencer. ^b^ Number of remaining paired reads after the trimming step. ^c^ Percentage of surviving paired reads/Raw Paired Reads. ^d^ Trinity has created a list of transcripts (contigs). The transcripts are grouped in components loosely representing genes. Transcript names are prefixed by the component/gene name, e.g., transcripts c115_g5_i1 and c115_g5_i2 are derived from the same isolated de Bruijn graph and therefore share the same component/gene number c115_g5. ^e^ Corresponding contig length distribution N50 = 4052 bp (Figure 4). ^f^ Corresponding contig length distribution N50 = 5512 bp.

**Table A2:** Top 25 up-regulated transcripts of *Inonotus obliquus* library in library of cells cultivated in presence of betulin.

Rank	Log2 FC	Description	E-Value	Accession
1	1.66	Phosphatidylinositol 4-phosphate 3-kinase	1.0	O70173.1
2	1.65	Alpha-ketoglutarate-dependent xanthine dioxygenase	3 × 10^−28^	Q4QZZ9.1
3	1.59	Probable lysosomal cobalamin transporter	0.79	Q8K0B2.1
4	1.57	Major vault protein beta	0.53	P54659.1
5	1.48	Diphthine methyl ester synthase	9.8	Q7S949.1
6	1.46	ATP-dependent RNA helicase DEAH12	7 × 10^−38^	F4KGU4.1
7	1.44	No hit		
8	1.44	No hit		
9	1.43	No hit		
10	1.42	No hit		
11	1.40	Superoxide dismutase [Mn]	8.2	Q9UQX0.1
12	1.40	Orotate phosphoribosyltransferase	6.1	A6VLF2.1
13	1.33	No hit		
14	1.32	Caffeic acid 3-O-methyltransferase	1.5	Q43239.1
15	1.32	No hit		
16	1.31	30S ribosomal protein S7	0.23	B3EUF4.1
17	1.29	No hit		
18	1.29	No hit		
19	1.28	No hit		
20	1.28	No hit		
21	1.27	No hit		
22	1.27	D-xylulose reductase	2 × 10^−13^	Q07993.1
23	1.27	No hit		
24	1.26	No hit		
25	1.26	No hit		

**Table A3:** Top 25 down-regulated transcripts of *Inonotus obliquus* library in library of cells cultivated in presence of betulin.

Rank	Log2 FC	Description	E-Value	Accession
1	−1.61	Isoleucine-tRNA ligase	1 × 10^−42^	Q21926.1
2	−1.55	N-acetyltransferase eso1	2 × 10^−20^	O42917.1
3	−1.52	Probable inactive purple acid phosphatase 16	0.47	Q9SR79.1
4	−1.48	No hit		
5	−1.45	ATPase family AAA domain-containing protein 1	2 × 10^−52^	F6QV99.2
6	−1.44	E3 SUMO-protein ligase NSE2	1 × 10^−8^	Q7ZXH2.1
7	−1.42	No hit		
8	−1.42	Cyclin-dependent kinase 1	0.21	P04551.1
9	−1.42	H/ACA ribonucleoprotein complex subunit NHP2	3 × 10^−48^	P32495.2
10	−1.40	Structural maintenance of chromosomes protein 1	2 × 10^−31^	O94383.2
11	−1.39	No hit		
12	−1.38	No hit		
13	−1.38	Tyrosine-protein kinase receptor ver-4	1.8	Q21041.2
14	−1.37	No hit		
15	−1.37	Proteasome-activating nucleotidase 2	2.4	Q5UT56.1
16	−1.37	Structural maintenance of chromosomes protein 2	0.0	P41003.2
17	−1.36	Structural maintenance of chromosomes protein 6	3 × 10^−159^	P53692.1
18	−1.36	Condensin complex subunit 3	4 × 10^−110^	Q10429.1
19	−1.36	No hit		
20	−1.36	Deoxynucleoside triphosphate triphosphohydrolase	1 × 10^−102^	Q0VCA5.1
21	−1.35	NFX1-type zinc finger-containing protein 1	5 × 10^−42^	Q8R151.3
22	−1.35	Cysteine-rich receptor-like protein kinase 41	0.001	O23081.2
23	−1.34	U3 small nucleolar RNA-associated protein 3	0.40	Q12136.1
24	−1.34	No hit		
25	−1.33	H/ACA ribonucleoprotein complex subunit cbf5	9 × 10^−102^	O43102.1

**Table A4:** Top 25 up-regulated transcripts of *Inonotus obliquus* library of cells cultivated in presence of white birch bark.

Rank	Log2 FC	Description	E-Value	Accession
1	6.09	Phosphatidylinositol 4-phosphate 3-kinase	1.0	O70173.1
2	5.89	Major vault protein beta	0.53	P54659.1
3	5.89	No hit		
4	5.75	DNA-directed RNA polymerase subunit beta	1.1	Q0ANP4.1
5	5.75	No hit		
6	5.71	Acetyl-CoA acetyltransferase IB	2.0	Q04677.3
7	5.70	No hit		
8	5.70	Orotate phosphoribosyltransferase	6.1	A6VLF2.1
9	5.67	Methyl-CpG-binding domain-containing protein 11	2.5	Q9LW00.1
10	5.64	F-box DNA helicase 1	2.6	F1ND48.2
11	5.62	No hit		
12	5.59	No hit		
13	5.58	No hit		
14	5.56	Glutamyl-tRNA reductase	1.5	A4SV44.1
15	5.54	No hit		
16	5.53	Segregation and condensation protein A	4.5	P47455.1
17	5.49	Nascent polypeptide-associated complex subunit beta	1.2	A5DF06.2
18	5.48	No hit		
19	5.48	Glucose-6-phosphate isomerase	0.39	Q83XM3.1
20	5.47	Caffeic acid 3-O-methyltransferase	1.5	Q43239.1
21	5.44	No hit		
22	5.43	No hit		
23	5.43	Protein AegA	2.7	P37127.2
24	5.43	No hit		
25	5.43	No hit		

**Table A5:** Top 25 down-regulated transcripts of *Inonotus obliquus* library of cells cultivated in presence of white birch bark.

Rank	Log2 FC	Description	E-value	Accession
1	−5.03	No hit		
2	−4.91	Tryptophan synthase alpha chain	4.6	P50382.2
3	−4.30	No hit		
4	−4.15	No hit		
5	−3.98	No hit		
6	−3.76	No hit		
7	−3.75	No hit		
8	−3.74	No hit		
9	−3.71	No hit		
10	−3.69	GTP-binding protein EngA	7.9	Q3AAU6.1
11	−3.67	No hit		
12	−3.65	Uncharacterized protein C12orf29 homolog	6.0	Q8BHN7.1
13	−3.60	Protein numb homolog	0.33	P49757.2
14	−3.56	No hit		
15	−3.56	No hit		
16	−3.53	No hit		
17	−3.53	Leucine-rich repeat transmembrane protein FLRT1	5.7	Q6RKD8.2
18	−3.51	Manganese peroxidase H4	1 x 10^−132^	P19136.1
19	−3.51	No hit		
20	−3.50	L-ascorbate peroxidase, cytosolic	3.0	P48534.2
21	−3.45	No hit		
22	−3.44	No hit		
23	−3.43	DNA polymerase epsilon catalytic subunit A	1.7	Q9WVF7.3
24	−3.41	Putative lipase C4A8.10	0.001	O14162.2
25	−3.39	Glutamate racemase	3.4	P63638.1
